# Immunotherapy in colorectal cancer: current achievements and future perspective

**DOI:** 10.7150/ijbs.64077

**Published:** 2021-09-03

**Authors:** Ahui Fan, Boda Wang, Xin Wang, Yongzhan Nie, Daiming Fan, Xiaodi Zhao, Yuanyuan Lu

**Affiliations:** State Key Laboratory of Cancer Biology and National Clinical Research Center for Digestive Diseases, Xijing Hospital of Digestive Diseases, Fourth Military Medical University, Xi'an, Shaanxi 710032, China.

**Keywords:** colorectal cancer, immunotherapy, immune checkpoint inhibitors, adoptive cell therapy, cancer vaccines, biomarkers

## Abstract

Following dramatic success in many types of advanced solid tumors, interest in immunotherapy for the treatment of colorectal cancer (CRC) is increasingly growing. Given the compelling long-term durable remission, two programmed cell death 1 (PD-1)-blocking antibodies, pembrolizumab and nivolumab (with or without Ipilimumab), have been approved for the treatment of patients with metastatic colorectal cancer (mCRC) that is mismatch-repair-deficient and microsatellite instability-high (dMMR-MSI-H). Practice-changing results of several randomized controlled trials to move immunotherapy into the first-line treatment for MSI-H metastasis cancer and earlier stage were reported successively in the past 2 years. Besides, new intriguing advances to expand the efficacy of immunotherapy to mCRC that is mismatch-repair-proficient and low microsatellite instability (pMMR-MSI-L) demonstrated the potential benefits for the vast majority of mCRC cases. Great attention is also paid to the advances in cancer vaccines and adoptive cell therapy (ACT). In this review, we summarize the above progresses, and also highlight the current predictive biomarkers of responsiveness in immunotherapy with broad clinical utility.

## Introduction

Colorectal cancer (CRC) is the third most common cancer and the second cause of cancer-related death worldwide. It is considered as a global health issue with an urgent unmet need of new therapeutic strategies 1. Although screening has reduced the incidence and mortality, approximately 25% CRC patients present with advanced stage disease at the time of diagnosis, and in patients with early-stage disease nearly 25%-50% will develop metastasis 2-4. The patients with oligometastatic disease after tumor resection and systematic therapy have 5-year survival rates of 40%, whereas the patients with metastatic colorectal cancer (mCRC) are only about 20% 5-8. While the benefits of chemotherapy and targeted therapy have reached a plateau, it is urgent to develop new effective treatment strategy to improve survival outcome.

Immunotherapy is aiming at harnessing the immune system to battle cancer. Immune checkpoint inhibitors (ICIs), modulating the interaction of T cells, antigens-presenting cells (APCs) and tumor cells to help unleash suppressed immune responses, emerged as a very effective therapy for patients with mCRC that is mismatch-repair-deficient (dMMR) or microsatellite instability-high (MSI-H) (termed dMMR/MSI-H mCRC). Owing to the efficacious, stable and durable responses, pembrolizumab and nivolumab (with or without Ipilimumab) were approved by US Food and Drug Administration (FDA) for the treatment of these patients. However, mCRC is characterized by insufficient mutated tumor antigens 9, thus the main challenge is to provide the benefit of immunotherapy for the vast majority of mCRC patients that are mismatch-repair-proficient (pMMR) or microsatellite-stable (MSS) or low microsatellite instability (MSI-L) (termed pMMR/MSS/MSI-L mCRC). In this review, we summarize the present evidences supporting the use of ICIs in CRC, focus on the recent advances in expanding the scope of ICIs in pMMR/MSS/MSI-L CRC, sum up the research progress of other anticipated immuno-approaches and highlight the emerging biomarkers for prediction of responsiveness to immunotherapy.

## ICIs-based immunotherapy

### Clinical Application of ICIs

#### Biomarkers: MMR and MSI

Mismatch repair (MMR) plays a critical role in maintaining DNA fidelity 10. By quantification of the MMR proteins MLH1, MSH2, MSH6 or PMS2 using immunohistochmical staining, CRC can be divided into dMMR or pMMR CRC 11. Notably, the change of MMR status can contribute to the change of microsatellite length named microsatellite instability (MSI) due to insertions and deletions, which can be detected accurately by PCR or next-generation sequencing 11. In dMMR-MSI-H tumors, major histocompatibility complex (MHC) class I-peptide complexes present on the surface of tumor cells, including mutated peptides which could be recognized as neoantigens and subsequently promote immune cells priming and infiltration. In particular, CD8+ tumor-infiltrating lymphocytes (TILs), T helper 1 (TH1) CD4+T cells and macrophages migrate into the tumor microenvironment, and elicit IFNγ secretion and anti-tumor effect. Meanwhile, dMMR-MSI-H tumor cells continuously upregulate T cell inhibitory ligands to promote immune escape, such as PD-L1, CD80 and CD86 of the B7 family 12-16.

Nearly 15% of all CRCs are dMMR-MSI-H, and the percentage is associated with tumor stage 17. Approximately 5%~20% of stage 2 and 11% of stage 3 tumors are dMMR-MSI-H, whereas, the percentage is only 5% in stage 4 18. Moreover, dMMR-MSI-H is a prognostic biomarker for patients of different stages 18-21. In stage 2 and stage 3, patients with dMMR-MSI-H tumors have much better prognosis than those with pMMR-MSI-L tumors. Remarkably, stage 4 patients with dMMR-MSI-H show dismal prognosis, but have good response to immune checkpoint blockade 22.

#### Approval for the second-line treatment in dMMR-MSI-H mCRC

Pembrolizumab and nivolumab (with or without Ipilimumab) are clinically approved in 2017 as the second-line treatment for patients with dMMR-MSI-H mCRC. KEYNOTE016 (NCT01876511) is a phase II trial using pembrolizumab to treat patients with refractory mCRC 23. The overall response rate (ORR) and disease control rate (DCR) were 50% and 89% for dMMR-MSI-H mCRC compared with 0% and 16% for pMMR-MSI-L, respectively. CheckMate142 (NCT02060188) is a phase II trial using nivolumab, with or without ipilimumab in patients with dMMR-MSI-H mCRC 24. In a median follow-up duration of 13.4 months, 55% and 80% of 119 patients in the nivolumab monotherapy arm showed ORR and DCR of 12 or more weeks, respectively. The 12-month progression free survival (PFS) and overall survival (OS) were 71% and 85%, respectively 25, 26.

### Exploration of ICIs in dMMR-MSI-H CRC

Given the cogent efficacy of immunotherapy in dMMR-MSI-H mCRC, the interest in exploring the potential of immunotherapy for the first line treatment of CRC patients, even in earlier-stage, is increasingly growing. The promising data of several randomized clinical trials has elicited tremendous excitement 27-29.

#### First-line treatment

KEYNOTE177 trial has attracted great attention, as an international randomized phase III study to assess pembrolizumab monotherapy verse standard of care in previously untreated stage 4 dMMR-MSI-H CRC patients 30. The primary end points are PFS and OS, and the secondary end point is ORR. Practice-changing results supported first-line pembrolizumab monotherapy for dMMR-MSI-H mCRC 31. The PFS and ORR were 16.5 months and 43.8%, respectively, in the pembrolizumab group as compared with 8.2 months (hazard ratio 0.60) and 33.1%, respectively, in the combined chemotherapy and bevacizumab/cetuximab group. The median DOR in the control group is 10.6 months while it had not been reached in the pembrolizumab group. With respect to safety, the pembrolizumab group showed less treatment-related adverse events in patients with grade 3 or higher. Based on the compelling data, FDA approved pembrolizumab for first-line treatment of dMMR/MSI-H mCRC in Jun.2020. Additionally, after a median follow-up duration of 44.5 months (36.0-60.3) with pembrolizumab vs 44.4 months (36.2-58.6) with chemotherapy, final analysis demonstrated that the ORR of the pembrolizuman increased from 43.8% to 45%.

The efficacy and safety of first-line treatment with combined nivolumab and low-dose ipilimumab in patients of dMMR-MSI-H tumors were evaluated in CheckMate142 32. After a median follow-up period of 13.8 months, the ORR and DCR were 60% and 84%, respectively, and the rate of complete response (CR) was 7%. An updated analysis of patients followed-up for a median of 29.0 months presented in the 2020 demonstrated a similar DCR 84%. Notably, the rate of CR increased from 7% at 13.8 months to 13% at 29.0 months, and the ORR increased from 60% to 69% 32. Compared with pembrolizuman monotherapy in KEYNOTE177, the combination of nivolumab and ipilimumab showed better efficacy and safety.

Additionally, COMMIT (NCT02997228) is an undergoing randomized phase III trial of mFOLFOX6/bevacizumab combination with or without atezolizumab in 347 previously untreated patients with dMMR-MSI-H mCRC. The primary end point is PFS. The secondary end points are OS, ORR, DCR and incidence of adverse events 33. The results are highly anticipated.

#### Adjuvant and Neoadjuvant Therapy

Adjuvant chemotherapy is indispensable for stage 3 CRC. Aiming to evaluate the potential efficacy of immunotherapy in adjuvant treatment, ATOMIC (NCT02912559), an ongoing phase III randomized controlled trial, enrolled 700 patients with stage 3 dMMR-MSI-H colon cancer. The patients were divided into 2 groups, which received 6 months of FOLFOX or FOLFOX plus atezolizumab for 6 months followed by atezolizumab alone for 6 months, respectively 34, 35. The primary trial end point is disease-free survival (DFS), and the secondary end points include OS and incidence of adverse events. The results are highly anticipated.

High efficacy of neoadjuvant immunotherapy in early-stage CRC has been proved. NICHE (NCT03026140), a phase II exploratory study, enrolled 40 patients with stage 1-3 colon cancer, including 21 patients with dMMR tumors and 20 with pMMR tumors 36, 37. The primary endpoint is safety and feasibility. All patients with dMMR tumors were treated with a single dose of ipilimumab and two doses of nivolumab, successfully received surgery on schedule and met the primary endpoint. The exciting results showed that all of the 20 patients with dMMR tumors achieved pathological responses, with 19 major pathological responses (MPR) and 12 pathological complete responses (pCR).

NRG-GI002 (NCT02921256) is a randomized phase II trial to study the efficacy of veliparib or pembrolizumab when combined with chemotherapy and radiation therapy in patients with locally advanced rectal cancer (LARC) 38. The primary endpoint is change in neoadjuvant rectal cancer (NAR) score, and the key secondary endpoints include OS, DFS, toxicity, pCR, clinical complete response (cCR), and sphincter sparing surgery (SSS). 185 patients were randomized to pembrolizumab (n= 90) or control (n=95) group 39. No improvement in NAR score was observed. The pCR was 29.4% in the pembrolizumab group compared with 31.9% in the control group, with the cCR 13.6% vs 13.9%. The PFS and OS have not been reached.

VOLTAGE (NCT02948348) is a phase I b/II open-label single-arm study to investigate the safety and efficacy of sequential use of neoadjuvant immunotherapy after chemoradiotherapy with capecitabine and subsequent surgery in patients with locally advanced resectable rectal cancer 40. 3 of 5 patients with dMMR-MSI-H tumors achieved pathological complete response and major pathological response 41.

Hopefully, neoadjuvant immunotherapy has the potential to become standard therapy of dMMR-MSI-H CRC in the near future.

### Exploration of Immunotherapy in pMMR-MSI-L CRC

Unlike dMMR-MSI-H CRC, pMMR-MSI-L tumors, which contribute to 95% of all mCRC cases, harbor a much lower mutation burden and poor recruitment of immune cells, leading to an unsatisfactory response to ICIs. With the deepening understanding of tumor mircoenvirnment of CRC, new discoveries and strategies of immune modulation have been explored and tested in pMMR-MSI-L mCRC patients to overcome primary ICI resistance.

#### Combination of ICIs

Combination of PD-1/PD-L1 and CTLA-4-blocking antibodies has the potential to offer synergistic benefits to patients 42. CCTG CO.26 is a phase II trial of durvalumab (PD-L1 inhibitor) plus tremelimumab (CTLA-4 inhibitor) versus best supportive care (BSC) alone for patients with advanced refractory colorectal cancer (rCRC) (NCT02870920), aiming to evaluate the efficacy and safety of the ICIs combination 43. With a median follow-up of 15.2 months, the median OS was prolonged by 2.5 months (6.6 months for D+T+BSC vs 4.1 months for BSC). CCTG CO.26 is the first study suggesting that anti-PD-L1 plus anti-CTLA-4 may prolong the OS in patients with MSS rCRC.

Preclinical data suggested that the anti-tumor activity of ICIs may be enhanced by inhibiting PGE2 synthesis 44. In the exploratory study NICHE (NCT03026140), 4/15 (27%) pathological responses were observed in patients with pMMR tumors, who received ipilimumab plus nivolumab before surgery with or without celecoxib 45. CD8+PD-1+ T-cell infiltration was predictive of response in pMMR tumors.

Panitumumab, a monoclonal antibody (mAb) targeting the epidermal growth factor receptor (EGFR), is a standard therapy in KRAS/NRAS/BRAF wild-type mCRC, the resistance of which is associated with increased CTLA-4 and PD-L1 expression 46. LCCC1632 (NCT03442569) is a single-arm phase II clinical trial to investigate the safety and efficacy of nivolumab and ipilimumab with panitumumab in patients with KRAS/NRAS/BRAF wild-type MSS refractory mCRC 47. Among 49 evaluable subjects, 12-week response rate was 35%, with median PFS of 5.7 months 46. The results demonstrate the safety and efficacy of ICIs combined with anti-EGFR therapy in MSS mCRC, providing merits to further study. All above studies suggested that PD-1 plus CTLA-4 blockade could be a promising treatment strategy for patients with pMMR-MSS CRC, and further larger studies are warranted.

#### ICIs with Radiotherapy

Preclinical work highlighted that radiotherapy (RT) could cause immunogenic cell death (ICD), which subsequently cause release of damaged-associated molecular patterns (DAMPs), increase of antigen presentation by APCs, priming of T cells and antitumor effects via the abscopal effect 48. As a hallmark of ICD, DAMPs include cancer-associated neoantigens, inflammatory cytokines and upregulation of immunogenic cell surface markers on tumor cells and stoma.

Although interim results of a single arm phase II study (NCT02437071) reported only 1 of 22 patients of pMMR-MSI-L CRC responded to the combination therapy of pembrolizumab and external beam radiation 49, more encouraging results have been reported successively. In a phase II clinical trial (NCT03104439), dual blockade of CTLA-4 and PD-1 combined with RT yielded a DCR of 29.2% (7/24) and an ORR 12.5% (3/24), respectively 50. The short-term results of the VOLTAGE-A, a phase I b/II study (NCT02948348), suggested that neoadjuvant chemoradiotherapy (CRT) followed by nivolumab and radical surgery could effectively treat MSS patients with locally advanced rectal cancer (LARC) 41. Among 37 patients, one patient (3%) received clinical CR and refused radical surgery after aforementioned treatment, 11 (30%) patients had pCR (AJCC grade 0). The MPR (AJCC grade 0+1) rate was 38% (14/37). These cogent data indicated the huge potential of the treatment strategies of ICIs coupled with RT.

#### ICIs with MEK Inhibitor

Preclinical data suggested that the inhibition of MEK, a downstream effector of the RAS-MAPK pathway, increased tumor expression of MHC-I and PD-L1, stimulated clonal expansion of peritumoral T cells, and enhanced anti-tumor activity of ICIs 51, 52.

Accordingly, the combination strategy using the MEK inhibitor cobimetinib and the PD-L1 inhibitor atezolizumab was tested in a phase I b study (NCT01988896) 53, 54. Preliminary data reported in 2016 showed that 4 of 23 patients with CRC had a partial response (17%, 3 pMMR-MSI-L, 1 unknown). Follow-up results were presented in 2018, demonstrating manageable adverse events and partial responses in 7 of 84 patients with mCRC (8%; 6 MSS/MSI-L, 1 was MSI-H). Although potential synergistic effect was shown, such effect failed to be confirmed in a subsequent phase III study IMblaze370, which is a randomized trial (NCT02788279) using atezolizumab (with or without cobimetinib) versus regorafenib in patients with pMMR-MSI-L rCRC 55. To adress such unexpected failure, researchers pondered to modify the details and approaches of the combination strategy 56. Combined MEK inhibitor with ICIs is being tested in several trials 28, 57, 58.

#### ICIs with anti-angiogenic agents

Preclinical data suggested that anti-angiogenic agents could increase CD8+ T cell infiltration and enhance the anti-tumor activity of CD8+ T cell by upregulating the expression of PD-L1, reducing immunosuppressive cells (TAMs, Tregs), and enhancing interactions between APCs and dendritic cells 59-61.

Based on this theory, a phase I b study (NCT01633970) showed promising results. Of 14 patients with pMMR/MSI-L rCRC received ICI plus anti-angiogenic agents (atezolizumab + bevacizumab), 1 patient had an objective response and 9 patients had stable disease 62, 63. Recently, encouraging anti-tumor activity from the combination therapy of regorafenib and nivolumab was further confirmed 64. In REGONIVO (NCT03406871), a phase I b trial, 25 patients with CRC (24 pMMR-MSS, 1 dMMR-MSI-H) were enrolled to examine the safety and efficacy of combination of nivolumab and regorafenib. Exciting results demonstrated an ORR of 36%. Median PFS was 7.9 months in CRC, with one-year PFS and OS rate 41.8% and 68.0%, respectively. Based on the encouraging data, investigations of larger cohorts are needed.

In LEAP-005 (NCT03797326), a nonrandomized, open-label, phase II study, the efficacy and safety of treatment with combined pembrolizumab and Lenvatinib in patients with previously treated advanced non-MSI-H/pMMR colorectal cancer were preliminarily evaluated 65. Among 32 patients with a median follow-up of 10.6 months, the ORR and DCR were 22% and 47%, respectively, with 2.3-month median PFS and 7.5-month median OS. The median DOR has not been reached. Based on the promising antitumor activity and a manageable safety profile, enrollment was expanded to 100 patients.

#### ICIs with Bispecific Antibodies

Bispecific antibodies are a new class of targeted therapeutics designed to bind two different sites on one antigen or two antigens. By targeting two different antigens,it simultaneously bridges the tumor cell and the T cell, hence enhance the intertumoral T cell infiltration and activation. CEA-CD3 is the first reported bispecific antibody showing significant efficacy in MSS CRC 66. CEA-CD3 plus atezolizumab (NCT02650713) revealed more effective clinical activity in patients with metastatic MSS CRC than CEA-CD3 monotherapy (NCT02324257) 67, 68. In the combination therapy group, the PR was 18% (2/11), and DCR was 82% (9/11) 69.

Another 3 emerging bispecific antibodies, TRAILR2-CDH17 (BI 905711) GCC-CD3 (PF-07062119) and CD137-PD-L1 (FS222), all showed potent T-cell mediated anti-tumor activity in CRC in preclinical trials 70-72. TRAILR2-CDH17 (BI 905711) was advanced into a Phase I a/b clinical trial for patients with advanced gastrointestinal cancers (NCT04137289) in 2019 73. As a novel strategy, bispecific antibody needs further investigation.

#### Other Prospective Combination

There are some alternative approaches of immune modulation worthy of high anticipation. One promising strategy is combining the immunotherapy with cancer vaccines, which may augment the host antitumor immune response 74. In a phase II study (NCT02981524) using GVAX colon vaccine (with cyclophosphamide) plus pembrolizumab, biochemical responses (≥30% decline in CEA) were observed in 7/17 (41%) patients of pMMR CRC, although it did not meet the primary objective 75. Continual expansion cohorts are ongoing, and more solid results are anticipated.

Oncolytic viruses (OVs) can cause direct lysis of tumor cells and promote anti-tumor immune response by inducing immunogenic cell death 76. As the OVs infection can turn the tumor from “cold” to “hot”, it enhances the anti-tumor capacity of the ICIs. The efficacy of the combination of OVs with ICIs has been preliminarily confirmed in clinical trials of a wide range of solid tumors 77-80. In a phase I clinical trial (NCT02636036), enadenotucirev (a chimeric adenovirus) plus nivolumab in solid tumors including CRC is currently being tested 81.

Intestinal bacteria play crucial roles in various fundamental physiopathologic processes 82-84. Intriguingly, it was recently found that intestinal bacteria Bifidobacterium pseudolongum could significantly affect the efficacy of ICIs, via releasing inosine and subsequently enhancing T cells activity 85. Remarkably, in anti-PD-1-refractory metastatic melanoma, the safety and feasibility of the combination of fecal microbiota transplantation (FMT) with ICIs were preliminarily confirmed in a phase I trial (NCT03353402) 86. These interesting discoveries could probably provide a new ideal strategy for patients with pMMR-MSS CRC.

## Cancer vaccines

Although existing for more than a century, cancer vaccines have barely received response in patients with CRC. Recently, cancer vaccines have elicited renewed interest owing to the convinced efficacy of immunotherapy. Multiple trials aiming to find the right antigenic stimulants are under investigation.

PolyPEPI1018 consists of 6 synthetic peptides with 12 unique epitopes derived from 7 conserved cancer testis antigens (CTAs) and optimized to induce long lasting CRC specific T cell responses 87. In OBERTO (NCT03391232), PolyPEPI1018 was tested in 11 previous untreated patients with MSS mCRC as an add-on to maintenance therapy. 4 patients achieved objective response and/or durable clinical benefit. Broad anticancer immunity was successfully boosted by PolyPEPI1018 at both peripheral and tumor level 88. Given PolyPEPI1018 promotes infiltration of cytotoxic CD8+ TILs into the core tumor, further study is warranted to investigate the efficacy of combination of PolyPEPI1018 with ICIs in patients with MSS mCRC.

Guanylyl cyclase C (GCC), a membrane-spanning receptor synthesizing the second messenger cyclic GMP (cGMP), is normally restrictedly expressed by intestinal epithelial cells and a subset of neurons, but universally expressed by metastatic colorectal tumors 89-91. Ad5-hGCC-PADRE vaccine uses a replication-deficient human type 5 recombinant adenovirus (Ad5) as the vector with GCC fused to the PADRE to induce GCC-specific immune responses. The safety and efficacy were evaluated in a phase I trial (NCT01972737) that enrolled 10 patients with surgically-resected stage 1 or 2 colon cancer 92. The preliminary results showed that 1 patient (10%) had antibody response to GCC and 4 patients (40%) exhibited GCC-specific T-cell responses, without significant toxicities 93. Notably, GCC-specific T-cell response was exclusively cytotoxic CD8+, but not CD4+ helper T cells, which were eliminated by self-tolerance 93.

Autologous vaccine OncoVAX is an active specific immunotherapy, which utilizes the patient's own tumor cells containing all relevant tumor-associated antigens to activate the body's immune system to prevent tumor progression after surgery. A randomized phase IIIb trial is being conducted to evaluate the recurrence status of colon cancer following surgery in 500 patients with stage 2 colon cancer after treatment with OncoVAX (NCT02448173) 94.

## Adoptive cell therapy

Another highly anticipated novel treatment to stimulate tumor immunity is adoptive cell therapy (ACT). ACT selects either host cells exhibiting antitumor activity or host cells engineered with chimeric antigens receptors (CARs) or antitumor T cell receptors (TCR) to augment the host antitumor immune response 95. Both CAR T therapy and TIL (tumor-infiltrating lymphocyte) therapy have evoked encouraging preliminary results, but the applicability remains to be proved.

The levels of CEA are low or absent in normal cells, but abundant in CRC 96. Based on this, several trials targeted CEA for ACT. In a phase I trial, CAR T-cells therapy targeting CEA was firstly tested in 3 patients with mCRC 97. Obvious decreases in serum CEA was observed in all of patients, and one patient received objective response of lung and liver metastasis. Unfortunately, all 3 patients experienced severe colitis. Another phase I trial (NCT02349724) was conducted to evaluate the safety and efficacy of anti-CEA CAR-modified T cells in CEA positive refractory mCRC patients 98. 7 of 10 patients obtained stable disease, without significant CAR-related toxicity.

The shed natural killer group 2D (NKG2D) ligands from tumor cells may downregulate NKG2D expression on NK and T cells, contributing to tumor immune escape 99-101. A novel attempt to further augment the host antitumor immune response is to genetically modify CAR T cells to express proteins such as PD-L1 and NKG2D receptor. The safety and efficacy of this “armored” CRATs remain to be investigated.

Encouraging results on TILs were shown in a case report 102. Researchers identified a polyclonal CD8+ T-cell response against mutant KRAS G12D in TILs and transferred the TILs into the patient. The result showed that all 7 metastatic lung lesions regressed at the first follow-up of 40 days, and the patient had a 9-month partial response until one lesion had progression. The patient remained 4 months clinically disease-free after the lung resection.

## Biomarkers of Response to Immunotherapy

Immunotherapy has significantly changed clinical management of CRC. Nevertheless, there is urgent need to explore specific biomarkers to predict responsiveness of immunotherapy. Emerged biomarkers in CRC immunotherapy are classified into 4 main types: tumor mutations, pre-existing immune responses, PD-L1 expression and the microbiota. A limited number of candidate biomarkers in CRC are listed below.

### Tumor Mutations

#### Tumor mutational burden

Tumor mutational burden (TMB) refers to the total number of somatic mutations per coding area of a tumor genome, which can measure all non-synonymous coding mutations in a tumor exome 103, 104. TMB has been proved to be an independent predictor of therapeutic efficacy of ICIs in several solid tumors including CRC 105-107. As known by now, higher TMB is associated with stronger immunogenicity, which could probably enhance the anti-tumor activity of immunotherapies. Notably, a high TMB value could emerge not only with MSI-H, but also in MSS tumors. The efficacy of immunotherapy was preliminarily confirmed in MSS CRC patients with a high TMB value. In the exploratory analysis of REGONIVO trial, TMB was evaluated in 23 patients with CRC. ORRs were 50% and 35.3% in the TMB high and low group, respectively, and the median PFSs were 12.5 vs 7.9 months. Another CCTG CO.26 trial assessed the plasma TMB by analyzing the cfDNA in blood samples. Higher plasma TMB with a cutoff value of 28 per megabase is associated with better OS in MSS CRC patients subjected to the combined PD-L1 and CTLA-4 inhibition. It is suggested that plasma TMB ≥28 may be a biomarker of identifying patients most likely to benefit from durvalumab plus tremelimumab. Undoubtedly, TMB is a great promising biomarker and remains to be further investigated.

#### POLE/POLD1

*POLE* and *POLD1* are crucial for polymerase ε and δ encoding, respectively, which are essential for proofreading and fidelity in DNA replication 108, 109. The somatic or germline mutations in POLE and POLD1 lead to the pathogenesis of CRC via a DNA hypermutated phenotype 110, 111. Nearly 7.4% of CRCs harbor mutations in either POLE or POLD1 and 74% of tumors with POLE or POLD1 mutations were MSS or MSI-L 112. Among pMMR CRCs, POLE-mutant CRCs show prominently higher CD8+ lymphocyte infiltration, expression of cytotoxic T-cell markers and effector cytokines than POLE wild-type CRCs, with upregulated expression levels of PD-L1, PD-1 and CTLA-4, etc. 113. Considering the enhanced immunogenicity, POLE may become another acceptable effective biomarker similar to MMR/MSI in the near future. NCT03435107, NCT03827044, and NCT03150706 are underway to investigate the benefit of ICIs in POLE-mutant CRC 114-116.

### Pre-existing Immune Responses

Tumor infiltrating lymphocytes (TILs) are associated with improved survival in retrospective studies of CRC patients, particularly for cytotoxic CD8+ T cells 117. Density and location of the intertumoral T cells could have a better prognostic value for CRC patients compared with the classical TNM system 118. Immunoscore is a scoring system evaluating the density of CD3+ T cells and CD8+ T cells both in tumor center and the invasive margin based on standardized criteria. A phase II multicenter study (NCT04262687) is ongoing, aiming to assess the anti-tumor activity of ICIs in combination with chemotherapy and antiangiogenic agents as first-line treatment of pMMR-MSI-L mCRC with high Immunoscore 119.

Based on the concept of Immunoscore, the classification of tumors was redefined as hot, altered and cold, which are routinely used referring to T cell-infiltrated, inflamed but non-infiltrated, and non-inflamed tumors, respectively 120. The redefinition not only includes Immunoscore, but the tumor immune contexture and microenvironment. The hot tumor could be more sensitive to ICIs, therefore, patients with hot tumors could probably gain more benefits from the immunotherapy. Furthermore, cold tumor can be converted into hot by radiotherapy, chemotherapy and so on. It is incredibly enlightening to develop and validate strategies to overcome primary ICI resistance.

### PD-L1 expression

PD-L1, a co-inhibitory receptor ligand, is recognized as one of the most widely studied biomarkers assayed by immunohistochemical staining, while PD-L1 expression status has not proved to be associated with the efficacy of ICIs in CRC to date. In KEYNOTE016 (NCT01876511), a phase II trial investigating pembrolizumab in patients with refractory mCRC, PFS or OS appeared irrespective of PD-L1 expression level 121. In Checkmate142 (NCT02060188), another phase II trial investigating the efficacy of monotherapy of nivolumab and combination of nivolumab with ipilimumab, no significant association was found between PD-L1 expression and ORR 122.

### The Gut Microbiota

Gut microbiota have large effect on the efficacy of immunotherapies in multiple cancers, and the composition of the intestinal microbiome could be a potential predictor of the efficacy of ICIs 82-85. Bifidobacterium pseudolongum, A. muciniphila, Lactobacillus johnsonii and Olsenella species proved to be useful for enhancing the anti-tumor effect of ICIs therapy. Bifidobacterium pseudolongum and A. muciniphila were found to enhance the anti-tumor effect of ICIs therapy through Inosine-A_2A_R signaling 85. T cell-specific A_2A_R signaling could be a promising pathway, through which the gut microbiota shows synergistic effect with immunotherapies. Further studies investigating the mechanisms of the gut microbiota regulating host antitumor immune response are needed.

## Conclusions

Undoubtedly, great progress has been advanced in CRC immunotherapy over the past few years. Recently, FDA granted approval to pembrolizumab and nivolumab (with or without Ipilimumab) for the second-line treatment of patients with dMMR-MSI-H mCRC based on the cogent data from 2 phase II trials. Based on the compelling data of KEYNOTE177, FDA approved pembrolizumab for the first-line treatment of this subgroup in 2020. Multiple trials exploring potential benefit of ICIs as the first-line treatment for patients with dMMR-MSI-H CRC and those with early-stage dMMR-MSI-H CRC are underway and highly anticipated.

However, the real critical challenge is to find ways to overcome immunotherapy primary resistance in the vast majority of patients with pMMR-MSI-L mCRC. To modulate immune cells and enhance the therapeutic efficacy, various ICIs-based strategies were tested in this subgroup, such as combinational therapy with antibodies blocking PD-1 and CTLA-4, ICIs combined with radiotherapy, ICIs combined with small molecule TKIs such as MEK inhibition and anti-angiogenic agents, and ICIs combined with bispecific antibodies. Although several early-phase trials presented promising data, further studies are warranted to validate the safety and efficacy. Alternative attempts to stimulate tumor immunity and enhance anti-tumor activity including ACT and cancer vaccines are promising areas of active research.

Moreover, biomarkers-based treatment is the inevitable trend of immunotherapy. Selection criteria are indispensable to identify patients who may benefit from these agents. Although some of aforementioned promising biomarkers emerged, discovery and validation of sensitive and specific biomarkers remain as an extremely active area of investigation.

With insights gained from additional trials aiming to develop effective therapeutic strategies, novel combination and biomarkers will likely help to guide clinicians towards a more personalized treatment for CRC patients. It is reasonable to believe that immunotherapy may soon change the treatment landscape for CRC.

## Figures and Tables

**Figure 1:**
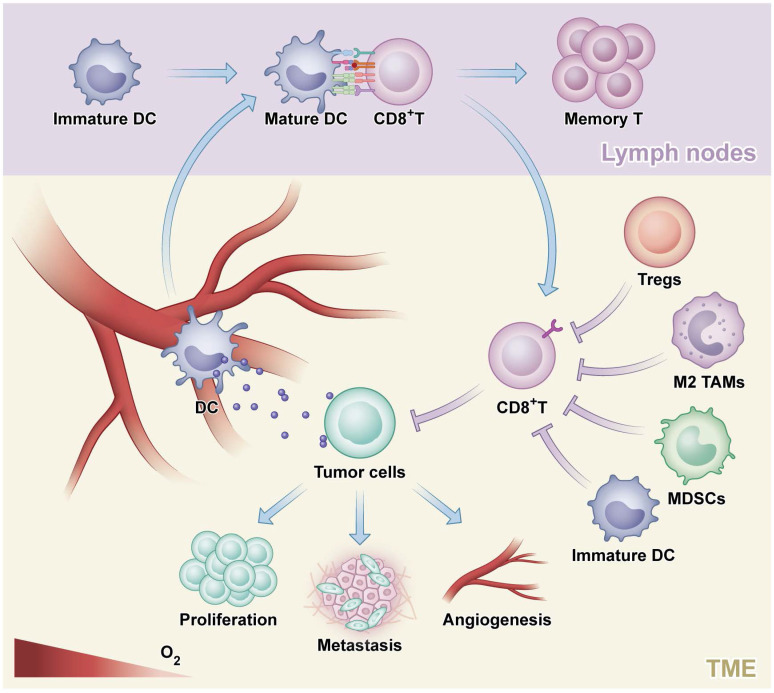
** The process of an antitumor response.** Tumor cells generate and release neoantigens which could be phagocytosed by dendritic cells (DC). Next, DC mediated presentation of tumor-specific antigens to CD8+ T cells and CD4+ helper T type1 cells which could enhance the effects of CD8+ T cells on killing tumor and promote the generation of tumor-specific activated T cells and memory T cells. Finally, tumor cells are destructed by effector T cells, which could be inhibited by regulatory T cells (Tregs), M2-polarized tumor-associated macrophage (M2 TAMs), myeloid-derived suppressor cells (MDSCs) and immature DC.

**Figure 2:**
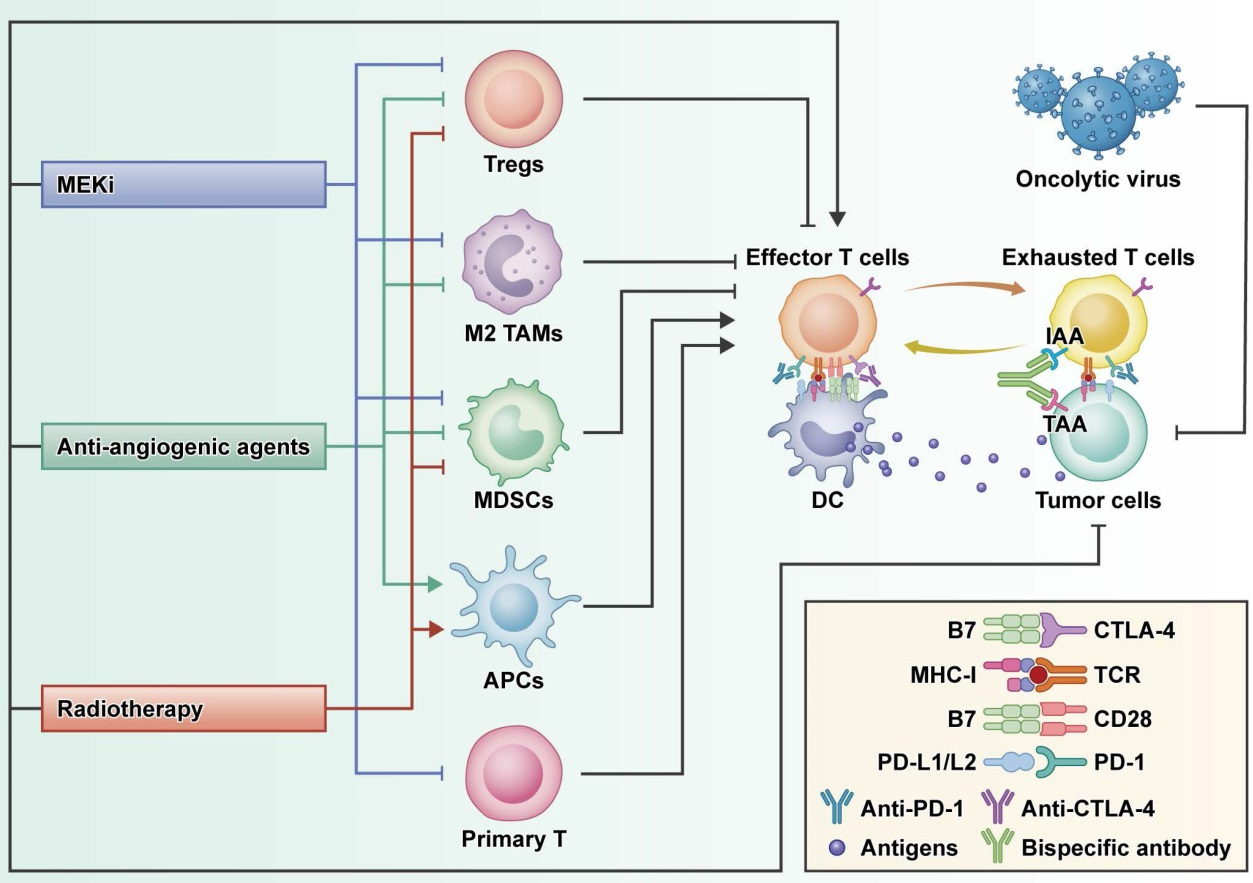
** The rationale of combination strategies to overcome primary ICI resistance in CRC.** MEKi: MEK inhibitor*;* Tregs: regulatory T cells; TAMs: tumor-associated macrophage; MDSCs: myeloid- derived suppressor cells; APCs: antigen presenting cells; DC: dendritic cells; IAA: immune-associated antigen; TAA: tumor-associated antigen.

**Figure 3:**
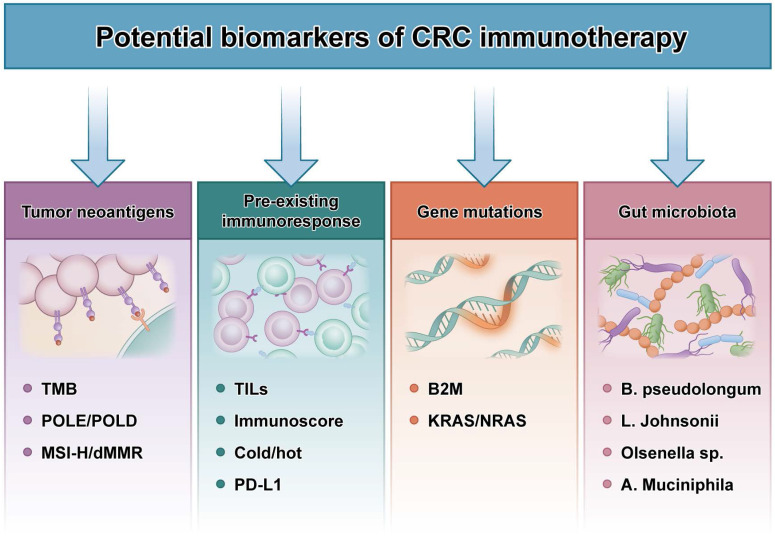
** Potential biomarkers of CRC immunotherapy.** TMB: tumor mutational burden; MSI-H: microsatellite instability-high; dMMR: mismatch-repair-deficient; TILs: tumor infiltrating lymphocytes; B2M: beta-2-microglobulin; B. pseudolongum: Bifidobacterium pseudolongum; L. johnsonii: Lactobacillus johnsonii; Olsenella sp.:Olsenella species; A. muciniphila: Akkermansia muciniphila.

**Table 1:** Ongoing trials in dMMR-MSI-H CRC

Treatment	Clinicaltrials.gov Identifier	Phase	Study treatment groups	Primary endpoint	Recruitment status
First-line	NCT02563002	III	Pembrolizumab versus standard-of-care chemotherapy	PFS, OS, ORR	Active, not recruiting
	NCT02060188	II	Nivolumab ± ipilimumab or daratumumab or anti-LAG3 antibody	ORR	Active, not recruiting
Adjuvant	NCT02912559	III	Adjuvant atezolizumab + FOLFOX versus FOLFOX alone	DFS	Recruiting
Neoadjuvant	NCT03026140	II	Nivolumab + ipilimumab ± celecoxib	Safety	Recruiting
	NCT02948348	Ib/II	Capecitabine + radiation + nivolumab + surgical therapy	pCR	Unknown
	NCT02921256	II	Pembrolizumab/ veliparib + chemotherapy + radiotherapy	NAR score	Active, not recruiting

PFS, progression-free survival; OS, overall survival; ORR, overall response rate; DFS, disease-free survival; pCR, pathological complete response; NAR, Change in neoadjuvant rectal cancer (NAR) score.

**Table 2:** Combination trials in pMMR-MSI-L CRC

Combination	Clinicaltrials.gov Identifier	Phase	Checkpoint inhibitors	Other intervention/ treatment (target)	Primary endpoint	Recruitment status
Radiation	NCT02888743	II	Duvalumab (PD-1) + Tremelimumab (CTLA-4)	-	ORR	Active, not recruiting
	NCT03007407	II	Duvalumab (PD-1) + Tremelimumab (CTLA-4)	-	ORR	Completed
	NCT03104439	II	Nivolumab (PD-1) + Ipilimumab (CTLA-4)	-	DCR	Recruiting
	NCT04575922	II	Nivolumab (PD-1) + Ipilimumab (CTLA-4)	-	DCR	Not yet recruiting
	NCT04030260	II	Nivolumab (PD-1)	Regorafenib (Multikinase)	PFS	Recruiting
	NCT04535024	II	Sintilimab (PD-1)	Stereotactic Ablative Radiotherapy	ORR	Recruiting
MEK inhibitor	NCT02060188	II	Nivolumab (PD-1) ± Ipilimumab (CTLA-4)	Cobimetinib (MEK)	ORR	Active, not recruiting
	NCT03271047	I/II	Nivolumab (PD-1) ± Ipilimumab (CTLA-4)	Binimetinib (MEK)	DLTs/ORR	Active, not recruiting
	NCT03377361	I/II	Nivolumab (PD-1) ± Ipilimumab (CTLA-4)	Tramatinib (MEK)	AEs, SAEs, ORR	Active, not recruiting
	NCT02788279	III	Atezolizumab (PD-L1)	Cobimetinib (MEK) + Regorafenib (multikinase)	OS, PFS, OR	Completed
	NCT03428126	II	Duvalumab (PD-1)	Tramatinib (MEK)	MTD, OR	Active, not recruiting
Anti-angiogenic Agents	NCT01633970	I	Atezolizumab (PD-L1)	Bevacizumab (VEGF)	AEs, DLTs, MTD	Completed
	NCT03406871	I/II	Nivolumab (PD-1)	Regorafenib (multikinase)	RD, MTD	Active, not recruiting
	NCT04446091	II	Carilizumab (PD-1)	anti-angiogenic TKIs ± Irinotecan	ORR	Recruiting
Bispecific antibody	NCT02650713	I	Atezolizumab (PD-L1)	RO6958688 (CEA - CD3)	AEs, DLTs, MTD	Completed
	NCT03752398	I	Ipilimumab (CTLA-4)	XmAb23104 (ICOS - PD-1)	AEs	Recruiting
	NCT04429542	I	Pembrolizumab (PD-1)	BCA101 (EGFR -TGFβ)	AEs, SAEs, DLTs	Recruiting
Oncolytic virus	NCT02636036	I	Nivolumab (PD-1)	Enadenotucirev	SAEs, DLTs, MTD	Recruiting
	NCT03206073	I/II	Duvalumab (PD-1) + Tremelimumab (CTLA-4)	Pexa-Vec	AEs	Active, not recruiting
	NCT04301011	I/II	Pembrolizumab (PD-1)	TBio-6517	AEs, MTD, MFD	Recruiting
Cancer vaccine	NCT02981524	II	Pembrolizumab (PD-1)	GVAX + Cyclophosphamide	ORR	Completed
	NCT03050814	II	Avelumab (PD-L1)	Standard of care ± Ad-CEA	PFS	Active, not recruiting
	NCT03639714	I/II	Nivolumab (PD-1) + Ipilimumab (CTLA-4)	GRT-C901 + GRT-R902	AEs, SAEs, DLTs	Recruiting
Anti-EGFR	NCT03442569	II	Nivolumab (PD-1) + Ipilimumab (CTLA-4)	Panitumumab (EGFR)	ORR	Active, not recruiting

ICIs, immunotherapy checkpoint inhibitors; PD-1, programmed cell death 1; CTLA-4, cytotoxic T-lymphocyte-associated protein 4; ORR, overall response rate; DCR, disease control rate; PFS, progression-free survival; DLTs, dose-limiting toxicities; AEs, number of adverse events; SAEs, number of serious adverse events; PD-L1, programmed cell death-Ligand1; OS, overall survival; MTD, maximum tolerated dose; MFD, maximum feasible dose; RD, recommended dose.

**Table 3:** Exploring cancer vaccines in colorectal cancer

Vector	Clinicaltrials.gov identifier	Phase	Patient population	Treatment/Target	Primary endpoints	Recruitment status
DC	NCT01885702	II	MSI-H CRC	DC vaccine	Safety, feasibility	Active, not recruiting
	NCT00103142	II	mCRC	PANVAC-V+PANVAC-F+DC	Reference-free survival	Completed
	NCT00558051	I	mCRC	DC vaccine	Safety, feasibility	Completed
	NCT01671592	I	CRC	Apoptotic autologous tumor-αDC1	Adverse events	Completed
	NCT04147078	I	CRC	Neoantigen-primed DC	DFS	Recruiting
	NCT03730948	I	CRC	DC vaccine	Safety, immune response	Recruiting
Peptide	NCT03391232	I/II	mCRC	PolyPEPI1018	Adverse events	Completed
	NCT00228189	I/II	mCRC	CEA	Immune response	Completed
	NCT01461148	I/II	MSI-H CRC	FSP	Immune response	Completed
	NCT03689192	I	mCRC	ARG1-18, 19, 10	Adverse events	Recruiting
	NCT00641615	I	CRC	RNF 43-721	Safety	Completed
Virus	NCT01147965	I/II	Colon cancer	AD5[E1-, E2b-]-CEA(6D)	Safety	Completed
	NCT01972737	I	Colon cancer	Ad5-hGCC-PADRE	Adverse events, antibody response	Completed
	NCT00027354	I	mCRC	TRICOM-CEA(6D)	Safety, immune response	Completed

DC, dendritic cell; MSI-H, microsatellite instability-high; CRC, colorectal cancer; mCRC, metastasis colorectal cancer; DFS, disease-free survival; Ad5-hGCC-PADRE, Guanylyl Cyclase C (GCC)-encoding replication-d human type 5 recombinant adenovirus vaccine.
